# Going viral: How social and personal motivations drive emotional engagement and consumer online brand-related activities

**DOI:** 10.1371/journal.pone.0336907

**Published:** 2025-11-18

**Authors:** Thi Cam Tu Dinh, Yoonjae Lee

**Affiliations:** Department of Business Administration, Yeungnam University, Gyeongsan, Korea; Paris School of Business, FRANCE

## Abstract

In today’s digital marketing landscape, viral brand campaigns succeed when consumers actively engage with and share brand messages across their social networks. Building on social exchange theory, this study examined what motivates people to emotionally connect with these campaigns and participate in consumer online brand-related activities (COBRAs). An online survey of 452 participants was conducted to examine the relationship between seven motivations, emotional engagement as a moderator, and COBRAs. The findings revealed that social motivations including the desire to interact, follow trends, build community, and stay connected strengthen emotional engagement with branded content and encourage sharing behavior. Personal motivations also proved significant, as individuals’ needs for self-presentation, self-expression, and self-assurance shaped both their emotional engagement and participation in brand-related activities. This research advances theoretical understanding in viral marketing, particularly regarding emotional engagement and COBRAs, while offering valuable insights for marketers seeking to design campaigns that resonate emotionally and socially with audiences.

## Introduction

Social media has transformed how brands and consumers connect, creating unprecedented opportunities for ongoing dialogue and collaboration. Platforms like Facebook and Instagram now enable constant interactions that facilitate real-time feedback and joint content creation between brands and their audiences [1]. This evolving digital landscape has made viral campaigns an exceptionally powerful form of electronic word-of-mouth (eWOM). Viral marketing has transformed how brands leverage social networking sites (SNSs) to distribute product information strategically, with the ultimate goal of encouraging content sharing across users’ social networks [2].

At its core, this marketing approach thrives on diverse forms of user engagement including thoughtful commentary, purposeful sharing of news and authentic reviews of brand experiences. By crafting content that resonates with audiences and encourages sharing, these campaigns deliver tangible business results through increased sales and reduced marketing costs while forging authentic connections across diverse consumer segments [3]. Global brands including Unilever, Burger King, Nike, and Coca-Cola have demonstrated the significant potential of viral marketing through campaigns that spark natural consumer participation in both message sharing and content creation [4].

Research in viral marketing has made significant progress in understanding brand content characteristics and consumer experiences [5–7]. However, the personal and social factors that motivate consumers to actively participate in online brand-related activities (COBRAs) remain understudied in the current literature. Current research lacks comprehensive understanding of this emotional mechanism, particularly regarding how consumer feelings drive participation in brand viral campaigns.

This research addresses these gaps through the framework of social exchange theory. Building on social exchange theory, brand-consumer relationships evolve through repeated positive interactions where individuals evaluate potential exchange value based on their brand connections [8]. This process inherently involves emotions, which emerge from and respond to external conditions and events [9]. These emotional responses shape both exchange dynamics and relationship development. Within the social exchange theory framework, emotional engagement emerges as a key psychological reward, creating vital links between consumer motivations and their subsequent behaviors [10].

COBRAs provide significant value to brands by enhancing consumer engagement and extending campaign reach across networks. Understanding these behaviors requires deep insight into consumer motivations for participating in activities like sharing and discussing viral campaigns. Research has identified several key factors driving campaign virality, ranging from entertainment value to purposive benefits [6], alongside fundamental needs for social connection and individual development [5]. Further studies reveal how branded content experiences shape COBRAs through multiple dimensions including aesthetic appeal, practical utility, identity expression, social relationships, and entertainment value [7,11]. These findings point to referral behaviors in COBRAs being driven by both personal motivations like identity construction and social motivations such as interpersonal interaction and relationship building [12].

This research extends the COBRAs literature by presenting an integrated model that reveals how social and personal motivations drive consumer engagement through emotional connections with brand content. While previous studies have explored various aspects of brand content value and social media experiences, this research takes a novel approach by demonstrating how four distinct dimensions of social interaction and three dimensions of personal identity influence consumer engagement decisions. The findings emphasize that successful viral campaigns require brands to recognize and leverage both personal and social motivation factors. The research also highlights the crucial mediating role of emotional engagement in connecting these motivations to referral behaviors [13]. This research is noteworthy as one of the early studies exploring the dynamic relationship between viral campaigns and COBRAs, advancing both interactive marketing theory and providing practical strategies for brands to strengthen consumer relationships and foster meaningful engagement with branded content.

## Literature review and theoretical background

### Brand viral campaign and social exchange theory

Research in viral marketing established its strategic importance for distributing content through social networks. Studies reveal that effective viral campaigns succeed through multiple forms of consumer engagement, ranging from substantive commentary to authentic experience sharing, which together generate value through peer-to-peer communication [2]. Findings have demonstrated that viral campaigns drive marketing effectiveness primarily through their expanded reach and resource efficiency when compared to conventional marketing methods [3]. To understand these viral campaign dynamics, researchers have employed various theoretical frameworks to explain consumer engagement behaviors. Among these perspectives, social exchange theory has emerged as particularly valuable for understanding how individuals evaluate and participate in viral brand content sharing [1,5,14,15]. This theoretical lens provides insights into the underlying mechanisms that drive consumers’ decisions to engage with and share branded content.

Social exchange theory posits that individuals engage in exchanges based on their evaluation of anticipated costs and benefits within their social relationships [8]. This evaluation process encompasses both tangible and intangible factors that shape consumers’ decisions to share their perspectives in online environments. Research has identified several key motivational benefits that drive consumers to share brand messages, including the inherent enjoyment of sharing, opportunities for strengthening social bonds, achievement of specific goals, enhancement of social status, development of meaningful connections, experience of reciprocal interactions, and channels for personal expression [1,5,14,15].

Prior research has established these social and personal factors as two distinct but interconnected dimensions that shape consumer engagement with brand content [12]. This research explores the roles of social and personal motivation factors as fundamental drivers of emotional engagement in viral brand campaigns. As consumers fulfill these social and personal motivations through brand content interaction, emotional engagement emerges as a key outcome that strengthens the connection between campaigns and their audiences, thereby enhancing brand message dissemination.

Within viral marketing research, the role of emotions extends beyond just an outcome of motivation fulfillment. Emotions have emerged as crucial elements that shape sharing behaviors, particularly through the social sharing of emotional experiences [16]. Studies demonstrate that consumer responses to brand content can be triggered by emotional reactions, whether positive or negative. When consumers experience heightened emotional states regarding products or services, they naturally gravitate toward sharing these experiences with others through social channels [17]. Prior research examining emotions in social exchange contexts has primarily positioned emotions as outcomes rather than intermediary factors. This study addresses this limitation by investigating emotional engagement’s mediating role between viral campaign exposure and subsequent sharing behaviors.

### Brand campaign emotional engagement and COBRAs

Recent research has underscored the significance of consumer engagement as a key strategic priority in marketing [18,19]. Engaged consumers establish deeper interactions with a company’s products or services, fostering stronger relationships and providing businesses with valuable insights for predicting future performance [20]. In the context of social media, consumer engagement encompasses physical, cognitive, and emotional involvement with the platform [10,15]. Among these dimensions, emotional engagement stands out as particularly impactful, encompassing feelings such as importance, excitement, motivation, pride, and a sense of challenge related to the consumer’s interaction with an online platform [10].

Consumer emotional engagement is fundamentally driven by both social and personal motivation factors. Research demonstrates that social motivations profoundly influence consumer behavior through their role in developing interpersonal connections, reinforcing social identities, and deepening brand content engagement, ultimately creating meaningful group affiliations [21,22]. Personal motivations prove equally significant in shaping emotional connections with brands, as consumers demonstrate stronger engagement with content that resonates with their individual values and identity. This alignment strengthens their self-concept while enabling authentic self-expression within their social circles [11]. When social and personal motivations converge, they create a powerful catalyst for emotional engagement with viral brand campaigns, allowing consumers to achieve both social connection and personal validation through their branded content interactions [23].

The social media environment has transformed consumer engagement into a dynamic interplay of participation and sharing activities that fundamentally shape customer discourse and purchase patterns [13]. This evolution enables organizations to actively participate in and guide brand-related conversations. Research shows that consumers interact with brand content through multiple touchpoints, including social reactions, content sharing, and commentary engagement [24]. These interactions manifest across an engagement continuum, ranging from passive content consumption to active participation through user-generated narratives and experiences.

The COBRAs framework, developed by Muntinga, Moorman [11], provides a structured approach for understanding how consumers engage with brand content across social media platforms. This conceptual framework distinguishes three distinct levels of engagement that characterize online brand interactions. As research has shown, these engagement levels progress from passive observation to active content generation [25]. The first level, consuming, involves users who observe brand content without direct interaction, essentially functioning as content viewers. The second level, contributing, represents intermediate engagement where users interact with existing content through comments or shares, yet stop short of creating new material. At the highest level, creating, users actively generate and distribute original brand-related content, which then becomes available for consumption and engagement by others in the social network.

Within this research framework, COBRAs function as a vital construct by directly aligning with viral brand campaign objectives. The campaigns typically progress through distinct phases, beginning with reach aquisition that correlates with consuming behaviors. As campaigns evolve, they seek to elicit positive brand engagement that naturally extends their reach. These advanced stages correspond to contributing and creating behaviors, representing deeper levels of audience participation [22]. The COBRAs framework thus provides an effective lens for examining audience referral behaviors within viral brand campaigns.

### Motivational drivers and hypotheses development

Research has identified two fundamental drivers of brand-related content creation: personal and social motivations [12]. The personal dimension connects deeply with self-identity, as consumers engage with viral campaigns to reinforce and express their authentic selves [22]. The social dimension, alternatively, emerges from consumers’ innate desire to connect with others who share similar content interests, fostering meaningful community relationships and shared social identities [14]. While these motivational dimensions operate distinctly, their synergistic interaction proves crucial in driving brand content engagement. Personal motivations enable authentic self-expression, whereas social motivations cultivate community interaction and group identification. Together, these complementary forces create the foundation for meaningful engagement with brand content, ultimately driving sharing behaviors and content creation [22,26].

Through systematic analysis of prior research, this study identifies distinct components within each motivation type that drive consumer engagement with viral brand content [7,11]. For social motivation, four key dimensions emerge that capture the spectrum of social connections consumers seek through brand engagement, ranging from direct interpersonal interactions to broader community participation. For personal motivation, three fundamental dimensions are identified that reflect the core aspects of identity expression and validation in brand-related activities. These dimensions have been consistently supported across multiple studies as distinct yet interrelated aspects of consumer engagement with viral brand content [8].

#### Social motivations.

Research has demonstrated that social motivations play a crucial role in shaping consumer referral behaviors, with content sharing primarily driven by interpersonal connections and communication needs [27]. These sharing behaviors are enhanced when consumers find content personally relevant and emotionally engaging [28]. By transforming traditional one-way brand communications into dynamic, user-centered engagement, viral brand campaigns effectively leverage these social motivation factors [14,29]. Building on this understanding, this study examines four distinct dimensions of social motivation that drive sharing behavior: social interaction, bandwagon effects, community building, and social connectedness.

Social interaction in viral brand campaigns reflects the behavioral engagement of consumers with brand content. It captures how users actively respond to and communicate around campaign messages, such as sharing thoughts, commenting, or expressing opinions [8,15,30]. Through these interactions, consumers create meaningful exchanges with others who share similar interests, which enriches the diversity of content circulating within their networks and encourages ongoing participation [21]. Connecting users through campaign involvement creates real bonds that keep them engaged and encourage more organic, word-of-mouth spread.

Bandwagon effects in viral campaigns emerge from people’s natural tendency to follow what others are doing. When people see others actively engaging with brand content, they feel drawn to participate in similar ways [31]. This behavior becomes particularly noticeable in social media, where users regularly observe and respond to others’ interactions with content. Studies show that seeing others enjoy and engage with content creates a powerful motivation for people to join in and share similar experiences themselves [32,33].

Community building reflects people’s desire to create lasting relationships around shared interests and values in brand communities. These communities grow naturally as members work together, share experiences, and develop common goals that matter to them [15]. When people feel they belong to these communities, they become more emotionally invested in the content and platform. The connections formed through these communities create deeper, more meaningful engagement that extends beyond simple interactions.

Social connectedness represents emotional and relational dimension, capturing the sense of belonging, attachment, and perceived closeness users feel toward others in the brand community through their participation [18]. By integrating brand interactions into their social networks, consumers enhance existing relationships and foster deeper social ties [34]. These emotional bonds support greater engagement and content sharing, as users leverage shared experiences to reinforce connections with others [19,35,36].

The preceding discussion demonstrates that social factors play a fundamental role in shaping how consumers connect with brand content through their communication and interaction needs. These social dimensions ranging from direct interactions to broader community connections create meaningful pathways for consumer engagement with viral campaigns. Building on this understanding, this study proposes that social motivation factors significantly influence emotional engagement in brand viral campaigns.

Hypothesis 1: Social motivation factors, comprising (a) social interaction, (b) bandwagon effects, (c) community building, and (d) social connectedness, are positively associated with emotional engagement in brand viral campaigns.

#### Personal motivations.

Personal motivation encompasses self-presentation, self-expression, and self-assurance, which work together to influence how people engage with branded content [11]. Social media users actively engage with brands to develop their online presence in ways that reflect their ideal identity and strengthen their connections within brand communities [7]. As they interact with brand content, people naturally express their preferences and reveal aspects of their personality to their social networks.

Self-presentation motivates people to share and create brand-related content that showcases their desired image to others [11]. Users thoughtfully select and share content that helps create favorable impressions among their audience [37]. This careful curation of brand interactions drives deeper emotional engagement with viral campaigns and influences how people participate in brand-related activities.

Self-assurance grows when others in the community acknowledge and respond to the brand-related content people create, boosting their confidence and sense of worth [28]. People actively seek recognition and respect through their content sharing and creation, which strengthens their emotional connection to the brand [38]. The validation they receive from these interactions creates emotional responses that encourage continued participation in brand campaigns.

Self-expression through branding reflects how people use brands to communicate their authentic selves to others [39]. People naturally gravitate toward and engage more deeply with brands that align with their personal values and identity [40]. When they find brands that effectively express their self-concept, they become more invested in interacting with brand content [41]. This alignment between brand and personal identity enhances emotional engagement and encourages participation across different levels of brand activities.

**Fig 1:**
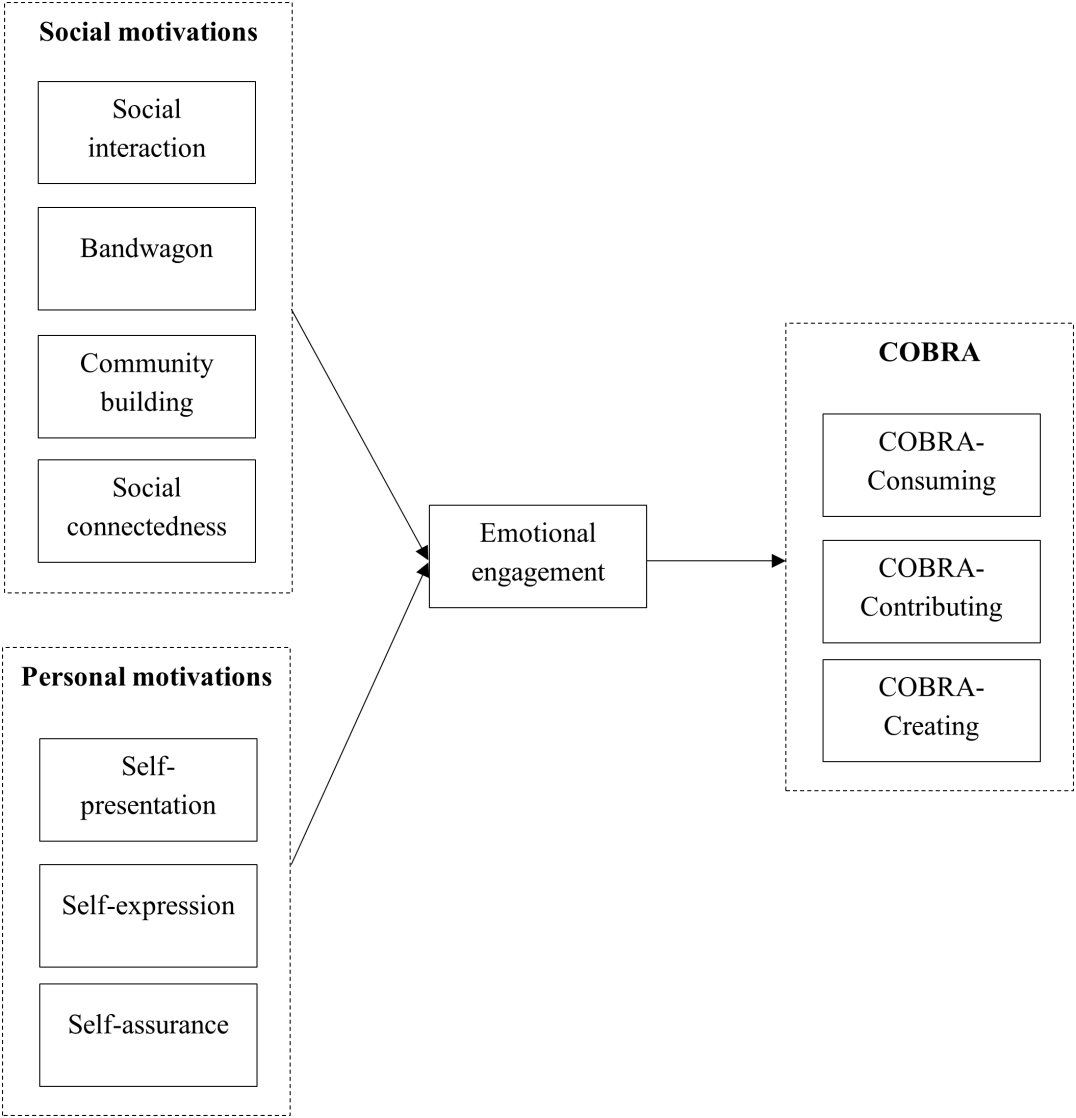
Research framework.

Personal motivations such as self-presentation, self-assurance, and self-expression primarily influence how individuals develop emotional engagement with brands. Rather than directly driving COBRAs, these motivations first increase the emotional connection that users feel toward brand content. Through this engagement, personal motivations can also drive different forms of COBRAs, which can even lead to seemingly passive forms of COBRAs, such as consuming, where users observe and internalize brand messages as part of their identity-related experience. Based on this understanding, we hypothesize as follows. Fig 1 presents the framework of this study.

H2: Personal motivation factors, comprising (a) self-presentation, (b) self-expression, and (c) self-assurance, are positively associated with emotional engagement in brand viral campaigns.

### Emotional engagement and COBRAs

People’s natural desire to share emotional experiences with their communities drives social sharing behavior in brand campaigns. This sharing requires significant engagement from users, characterized by positive experiences that emerge from their interactive and creative involvement with brands. Such engagement combines thoughtful consideration, emotional connection, and willingness to take action [42]. Research clearly shows the strong relationship between how people engage with brands and their participation in brand-related activities. Notably, Berger and Milkman [23] found that positive emotional responses serve as powerful catalysts for content sharing, highlighting how emotional engagement drives participation in consumer online brand-related activities.

Given these insights into the relationship between emotional engagement and COBRAs, this study examines how emotional engagement influences three distinct levels of participation in viral brand campaigns. Accordingly, we hypothesize as followings.

H3: Consumers’ emotional engagement in brand viral campaigns is positively associated with (a) consuming, (b) contributing, and (c) creating brand-related activities.

## Methodology

### Procedure

This study employed a self-administered online survey conducted on the Amazon Mechanical Turk platform in the US in 2024, as this platform has been widely used in previous research to gather participant opinions [43,44]. A pre-test with 50 participants was conducted prior to the main survey to ensure reliability. To maintain the validity of the survey, screening questions were included to filter out ineligible respondents. According to the annual report by Chaffey [45], the major age group for social media ranges from 18 to 50 years old. Thus, the initial screening affirmed participants’ ages fell within this scope. Additionally, participants needed past exposure to viral brand campaigns and could name at least one example encountered or interacted with. Participants were not exposed to campaign materials during the survey, instead, they were asked to recall a viral brand campaign from their own experience and reflect on their engagement with that campaign. This retrospective approach allowed for capturing authentic consumer experiences across diverse campaigns rather than restricting responses to a single stimulus.

After excluding respondents who did not meet the criteria, the final sample comprised 452 participants, with 191 women (42.04%) and 261 men (57.74%). Participants ranged in age from 19 to 49 years, with 1.11% aged 18–19, 38.72% aged 20–29, 44.25% aged 30–39, and 15.93% aged 40–49. Examples of popular viral brand campaigns cited by participants included Dove’s Real Beauty, Old Spice’s Smell Like a Man, and Nike’s Just Do it.

### Measurement

The constructs in this study were measured using scales adopted from previous research and adjusted to fit the context of this study. A seven-point Likert scale (1 = strongly disagree, 7 = strongly agree) was employed to assess all constructs. Social motivation was measured using the following scales: three items for social interaction from Jahn and Kunz [41], three items for bandwagon effects, four items for community building from Rathnayake and Winter [46], and three items for social connectedness from Chen [47]. For personal motivation, self-presentation was measured using scales from Jahn and Kunz [41] and De Veirman, Cauberghe [48], self-assurance using scales from Sjöblom and Hamari [49], and self-expression using scales from Rathnayake and Winter [46]. The brand campaign’s emotional engagement was assessed with three items adapted from Molinillo, Anaya-Sánchez [50]. Finally, COBRAs were measured using ten items from Buzeta, De Pelsmacker [25] to capture its three dimensions: consuming, contributing, and creating.

### Data analysis

The data in this study were analyzed using partial least squares structural equation modeling (PLS-SEM) with SmartPLS v3.2.9. PLS-SEM was chosen for its ability to handle models with multiple constructs and its flexibility in operating without strict assumptions about the normality of the data distribution [51]. This approach is particularly suitable for identifying key exogenous constructs within complex research models. Accordingly, PLS-SEM was employed to test the hypothesized relationships in this study.

## Result

### Confirmatory factor analysis

Confirmatory factor analysis (CFA) was conducted to assess the reliability, convergent validity, and discriminant validity of the constructs. Key metrics, including factor loadings, composite reliability (CR), average variance extracted (AVE), and the square root of AVE, were calculated. The results of these analyses are presented in Tables 1 and 2.

**Table 1:** Constructs’ items, reliability, and validity.

Constructs	Items	Factor loading	CR	AVE
**Social interaction**	I can meet people like me on the campaign	0.89	0.92	0.80
	I can interact with people like me on the campaign	0.88		
	I can meet people with my interests on the campaign	0.91		
**Bandwagon**	The campaign comforts me by letting me know the thoughts and opinions of others	0.91	0.92	0.79
	Seeing others’ activities on the campaign before I do helps me to avoid potential conflicts	0.84		
	I try to adjust my activities to the campaign posts based on others’ activities	0.91		
**Community building**	Campaigns help me to be part of a community that I would not otherwise have been part of	0.89	0.91	0.76
	Campaigns allow me to build a network that could bring me social support	0.84		
	Campaigns allow me to contribute to communities that make an impact on society actively	0.89		
**Social connectedness**	On campaigns, I feel I am connected to other users	0.88	0.91	0.76
	I have made connections to other people on the campaign	0.84		
	During the campaign, I feel comfortable communicating with other people	0.90		
**Self-presentation**	On the campaign, I can make a good impression on others	0.87	0.93	0.81
	On the campaign, I can improve the way I am perceived	0.92		
	On the campaign, I can present to others who I want to be	0.92		
**Self-assurance**	I like when other campaign participants take my activities into account	0.90	0.91	0.78
	I feel good when my activities prove to other campaign participants that I have knowledge about something	0.85		
	I try that my activities to improve my reputation among other campaign users	0.90		
**Self-expression**	The campaign allows me to express my opinions freely	0.90	0.91	0.77
	The campaign allows me to assert my identity freely	0.85		
	The campaign allows me to have my say	0.89		
**Brand campaign emotional engagement**	I am enthusiastic about the campaign.	0.80	0.90	0.75
	The campaign inspires me.	0.88		
	I am excited when participating in the campaign	0.91		
**COBRAs – Consuming**	I view the campaign content	0.90	0.91	0.77
	I watch the campaign content	0.85		
	I follow the campaign threads.	0.90		
**COBRAs – Contributing**	I engage in the campaign content or hot topics.	0.89	0.91	0.77
	I comment on the campaign -related microblogs, pictures, audio posts, videos, etc.	0.85		
	I retweet the campaign content.	0.88		
**COBRAs – Creating**	I publish the campaign-related content	0.89	0.93	0.76
	I write the campaign-related articles	0.87		
	I upload the campaign-related pictures, audio posts, or videos	0.86		
	I write reviews related to the campaign content	0.86		

**Table 2:** Discriminant validity and correlations.

	1	2	3	4	5	6	7	8	9	10	11
1. Social interaction	**0.90**										
2. Bandwagon	0.85	**0.89**									
3. Community building	0.85	0.85	**0.87**								
4. Social connectedness	0.87	0.86	0.87	**0.87**							
5. Self-presentation	0.86	0.83	0.84	0.87	**0.90**						
6. Self-assurance	0.85	0.87	0.85	0.87	0.85	**0.88**					
7. Self-expression	0.85	0.87	0.86	0.87	0.86	0.88	**0.88**				
8. Emotional engagement	0.85	0.86	0.85	0.86	0.86	0.86	0.86	**0.87**			
9. COBRAs – Consuming	0.79	0.83	0.80	0.81	0.80	0.85	0.85	0.82	**0.88**		
10. COBRAs – Contributing	0.79	0.80	0.81	0.81	0.81	0.83	0.82	0.80	0.86	**0.88**	
11. COBRAs – Creating	0.78	0.81	0.82	0.81	0.80	0.82	0.81	0.79	0.83	0.86	**0.87**

Note: Diagonals (ITALICS) represent the square root of the AVE, while the off-diagonals represent the correlations.

As shown in Table 1, all factor loadings exceeded 0.8, surpassing the recommended threshold of 0.7, confirming strong indicator reliability. Internal consistency was verified with a composite reliability (CR) value of 0.9. Convergent validity was assessed using the average variance extracted (AVE) to determine the extent to which the indicators correlate with their respective constructs. All AVE values exceeded 0.75, well above the threshold of 0.5, indicating strong construct alignment. Discriminant validity was also confirmed, as the square roots of all AVE values were greater than the corresponding inter-construct correlations, as detailed in Table 2.

### Structural model

The structural model was analyzed to test the study’s hypotheses by examining the t-values, p-values, and standardized path coefficients. Additionally, the mediation effects of brand campaign emotional engagement on the relationship between social and personal motivation factors and COBRAs were evaluated.

Table 3 indicates that all hypotheses were supported. The results revealed that social motivation factors significantly influenced emotional engagement (β = 0.132, p < 0.01). Similarly, positive relationships were observed between emotional engagement and bandwagon effects (β = 0.155, p < 0.01), community building (β = 0.129, p < 0.001), and social connectedness (β = 0.119, p < 0.01). These findings demonstrate that all four dimensions of social interaction motivations positively impact brand campaign emotional engagement, confirming the acceptance of H1.

**Table 3:** Hypothesis test.

Hypothesis	β	T	P	Result
H1a. Social interaction → Emotional engagement	0.132	2.616	0.009	Accepted
H1b. Bandwagon → Emotional engagement	0.155	2.922	0.003	Accepted
H1c. Community building → Emotional engagement	0.129	2.804	0.005	Accepted
H1d. Social connectedness → Emotional engagement	0.119	2.359	0.018	Accepted
H2a. Self-presentation → Emotional engagement	0.167	3.199	0.001	Accepted
H2b. Self-assurance → Emotional engagement	0.128	2.321	0.020	Accepted
H2c. Self-expression → Emotional engagement	0.148	2.844	0.004	Accepted
H3a. Emotional engagement → Consuming	0.823	36.334	0.000	Accepted
H3b. Emotional engagement → Contributing	0.803	35.45	0.000	Accepted
H3c. Emotional engagement → Creating	0.791	27.937	0.000	Accepted

Three paths were analyzed to examine the relationship between personal motivation factors and emotional engagement. The effect of self-presentation on emotional engagement was significant (β = 0.167, p < 0.05). Similarly, self-assurance positively influenced emotional engagement (β = 0.128, p < 0.05), and self-expression significantly influenced emotional engagement (β = 0.148, p < 0.001). These findings confirm the acceptance of H2, demonstrating that personal motivations are positively associated with emotional engagement in brand campaigns.

This study examined the impact of emotional engagement in viral campaigns on the three dimensions of COBRAs. The findings provided full support for H3, revealing a significant effect of emotional engagement on consuming (β = 0.823, p < 0.001), contributing (β = 0.803, p < 0.05), and creating (β = 0.791, p < 0.05).

Finally, the mediation effects of social and personal motivation on COBRAs were analyzed. The results indicated that all indirect paths were positively significant (p < 0.05), demonstrating that all factors of social interaction and personal identity motivations influence the three dimensions of COBRAs through the mediation of brand campaign emotional engagement.

## Discussion and conclusion

This study examined how social media users engage with viral brand campaigns, looking specifically at the roles that social and personal motivation factors, along with emotional engagement, play in shaping consumer behavior. The research findings supported our understanding of how these elements work together to influence how people interact with and share brand content.

The social aspects of viral campaigns emerged as powerful drivers of emotional engagement with brand content. People form stronger emotional connections when they can actively communicate and interact with others through the content [22]. This engagement intensifies as more people participate in the campaign, creating a bandwagon effect driven by both the fear of missing out and the natural desire to be part of group activities [52]. The research also showed that when brands successfully build communities around their content, people develop deeper emotional ties. These community connections enhance emotional well-being by creating meaningful social bonds through shared brand experiences [19,53].

Personal motivation factors proved equally important in building emotional connections with viral campaigns. When campaigns give people opportunities to express themselves authentically, they generate stronger emotional engagement. This aligns with previous findings about self-presentation in online spaces, where people actively share content that enhances their social image [37]. The research revealed that campaigns resonating with people’s personal values and beliefs create particularly strong emotional bonds [40]. Additionally, when campaigns help affirm people’s sense of self-worth and social position, they become more emotionally invested in the content.

The study found clear evidence that emotional engagement influences how deeply people involve themselves with brand content across all levels of participation. At the basic level, emotional connection drives people to consume content by watching, reading, and following campaign developments. As engagement deepens, people move beyond passive consumption to actively contribute through comments, shares, and reactions. At the highest level of involvement, strong emotional connections motivate people to create their own brand-related content, demonstrating their deep investment in the campaign. These patterns show how emotional engagement naturally propels people toward increasingly active forms of brand participation.

The findings of this study reveal that both social and personal motivations shape consumer engagement with brand viral campaigns through the mediating role of emotional engagement. Social motivations encourage people to connect with others, follow collective behaviors, and build communities around shared brand experiences. These social dynamics generate stronger emotional ties to campaign content, which in turn encourage people to consume, contribute to, and create brand-related activities. Personal motivations also play a critical role in allowing individuals to present themselves, gain confidence, and express their true identity, strengthening emotional connections with the content. Once these emotional bonds are established, they naturally lead consumers to participate in COBRAs at different levels, beginning with simple content consumption and extending to active contribution and creative involvement. These results show that social and personal motivations do not directly drive COBRAs but rather operate through emotional engagement, which acts as the bridge between consumer intentions and brand-related behaviors.

### Theoretical implications

This research enhances our understanding of viral marketing by revealing how social exchange dynamics shape digital brand interactions. The findings show that social exchange theory, traditionally focused on interpersonal relationships, offers valuable insights into why people engage with and share brand content on social media. By examining both social and personal motivations, the study illuminates the complex factors driving participation in viral campaigns.

The research particularly highlights emotional engagement as a crucial element in viral marketing success. Where previous studies often viewed emotional aspects as secondary outcomes, this investigation positions emotional engagement as the key driver connecting consumer motivations to active participation. This understanding helps explain how psychological rewards influence behavior in social media environments.

The findings address significant gaps in current marketing literature by demonstrating how social and personal factors work together to shape viral campaign engagement. While earlier research noted the importance of these motivations [5,22,41], this study reveals the specific mechanisms linking emotional engagement to consumer actions. The detailed examination of different social and personal dimensions shows how these elements combine to encourage varying levels of participation.

The research also provides new insights into how consumers progress through different stages of brand engagement. The findings show that emotional connections drive the transition from passive content consumption to active creation and sharing. This comprehensive view enhances our understanding of participation patterns in social media marketing, revealing how different elements work together to create successful viral campaigns.

### Practical implications

While effective communication remains a constant pursuit for brands, this research provides valuable insights for developing successful viral marketing initiatives. The investigation offers meaningful guidance for those seeking to craft increasingly influential communications. Firstly, it highlights the potential of initiatives to leverage consumer referral behaviors, enabling brands to amplify their messages effectively. By nurturing brand-related activities, organizations can transform customers into advocates, thereby extending campaign reach through social networks. This referral-driven approach proves particularly valuable, as consumers acquired through recommendations demonstrate higher loyalty and profitability [6]. To maximize such potential, marketing practitioners should focus on creating emotionally engaging content that inspires sharing and endorsement of brand messages among consumers.

This study highlights the fundamental role of both social and personal motivational factors in viral brand campaigns for developing emotional engagement. Marketing practitioners should design campaigns that facilitate meaningful social interactions among consumers through experience sharing opportunities. Strategic implementation of interactive elements such as polls and quizzes can naturally build connections within brand communities. The campaigns should also support individual expression by allowing consumers to showcase their unique identities. Customization features including personalized templates and branded creative assets serve two important functions. These features enable authentic self-expression while simultaneously encouraging content sharing behavior. Such an integrated approach effectively combines social and personal motivational factors to strengthen campaign effectiveness.

The findings underscore the critical role of emotions in shaping campaign success. Marketing practitioners must recognize how deeply emotional responses, particularly feelings of pride and personal enthusiasm, influence consumers’ emotional engagement with brand content. Research reveals that such emotional engagement acts as a powerful catalyst, naturally driving referral behaviors throughout social networks. The COBRAs framework proves particularly valuable for marketing practitioners seeking to assess campaign performance. This understanding allows marketing practitioners to measure not just surface-level metrics but meaningful engagement, from initial content consumption through active creation. To maximize such potential, marketing practitioners should focus on creating emotionally engaging content that inspires sharing and endorsement of brand messages among consumers.

## Limitation and future research

The scope of this research includes social and personal motivations and reveals a limitation in capturing the full range of factors that shape emotional engagement and consumer online brand-related activities in viral campaigns. Future research could investigate other dimensions, such as entertainment motivations, to better understand what drives consumer behavior in the context of viral marketing. Moreover, participants recalled viral brand campaigns they had previously encountered and reported their engagement. While this captures authentic experiences, it may introduce recall bias. Future studies could expose participants to campaigns directly to measure engagement in real time. Another limitation lies in the data collection, which limits the generalizability of the findings. This study explores opinions about brand viral campaigns and COBRAs in the US, so future studies could extend the scope by conducting cross-cultural research to determine whether the observed effects are consistent across different cultural contexts. Finally, the study did not control for factors such as prior brand affinity and frequency of social media use, which may also influence consumer engagement with viral campaigns. Future studies could account for these variables to provide a more nuanced understanding of the determinants of COBRAs.
